# Influence of Food on Recruitment Pattern in the Termite, *Microcerotermes fuscotibialis*

**DOI:** 10.1673/031.010.14114

**Published:** 2010-09-14

**Authors:** B.O. Olugbemi

**Affiliations:** Raw Materials Research and Development Council, Ondo State Office, P.M.B. 656, Akure, Ondo State, Nigeria

**Keywords:** Isoptera, Termitidae, termite behavior

## Abstract

Recruitment pattern in the termite, *Microcerotermes fuscotibialis* Sjostedt (Isoptera: Termitidae) was found to be largely influenced by the presence or absence of food. This is reflected in the quantitative recruitment that occurred after food had been detected by the scouting foragers. Results showed that this information was communicated and responded to by other confederates in the nest within four to seven minutes after food addition or removal. The presence of an additional food source relative to an already existing one did not have any significant impact on the recruitment activities of this termite. Recruitment decision pattern in *M. fuscotibialis* involve to a large extent an “autocratic” decision strategy in the development and maintenance of recruitment process in this termite species.

## Introduction

*Microcerotermes fuscotibialis* Sjostedt (Isoptera: Termitidae) is a wood-feeding termite, that constructs grayish-brownish narrow earthen runways, lined with fecal deposits over trees, walls of mud houses and wooden structures. Although they are subterranean termites, they build an ovoid or spherical arboreal carton nest with covered runways leading to the ground (Olugbemi and Malaka 1994).

According to Malaka and Leuthold (1986), many mechanisms have evolved in social insects, particularly in ants and termites, that function to assemble confederates for joint efforts in food retrieval and nest construction. Olugbemi and Malaka (2007) reported the utilization of pheromone to communicate the presence and location of food to other nest mates, which were subsequently recruited to the food site.

Wilson (1962) and Jaffe (1980) presented different models of decision making to explain different recruitment strategies of chemical mass recruitment to food in the ants. These models, as cited by Andara et al. (2004), were based on the following assumptions:

1. That the recruitment process of workers can be induced and regulated by chemical signals alone.
2. The number of workers leaving the nest is controlled by the amount of trail substance deposited by foragers.
3. That the trail pheromone orients the workers to the food source.
4. The more desirable the food find is, the more trail pheromone is presented to the colony, and hence, the more newcomer ants emerge from the nest.
5. The trail pheromone is a volatile substance, which if not reinforced, will evaporate below the threshold level at a certain time, depending on the initial concentration of the pheromone.

Thus the pattern of trail reinforcement is determined by the decision making system utilized during the recruitment process (Jaffe et al. 1985).

Foraging in termites is a social procedure (Traniello and Buscher 1985; Olugbemi and Malaka 1994), where the activities of hundreds or thousands of individuals are coordinated by trail pheromones, which stimulate foragers to leave the nest and orient them to a food find. Accordingly, a scouting termite returns to the nest after locating food in trail-laying posture, and discharging sternal gland pheromone (Kaib 1990).

Traniello et al. (1994) stated that exploratory activities for food are randomly carried out by workers of *Reticulitermes* species, with rewarded individuals returning to the nest in trail-laying postures. This subsequently resulted in the recruitment of other nest mates to the source of food. In an earlier study, Oloo and Leuthold (1979) observed that workers of *Trinervitermes bettonianus* also locate food by random exploration, with rewarded individuals communicating and recruiting other nest mates to the source of food.

Malaka and Leuthold (1986), found that there was quantitative recruitment in colonies of *Amitermes evuncifer* whenever food was discovered, thus stimulating the termites to accelerate their running speed to and from the food source. Olugbemi and Malaka (1994) obtained similar results for *M. fuscotibialis.*

In the Formosan subterranean termite, *Coptotermes formosanus*, search activity is adjusted in response to available food size (Hedlund and Henderson 1999). As food size increased, termite survival and food consumption increased, but search tunnel volume decreased. This activity led to fewer branching tunnels being excavated. According to Robson et al. (1995), termite foragers hunt for food in a systematic, branching pattern that was thought to minimize search redundancy, but once a food source is found, further tunneling activity is directed towards the food source (Campora and Grace 2001).

Results from the works of Andara et al. (2004) in the termites, *Nasutitermes ephratae* and *N. corniger* demonstrated the utilization of an “autocratic” rather than a “democratic” system in decision making during their recruitment processes. In this system, the termite scouts control the amount of pheromone deposited on a trail according to the quantity and quality of the food source.

**Figure 1.**
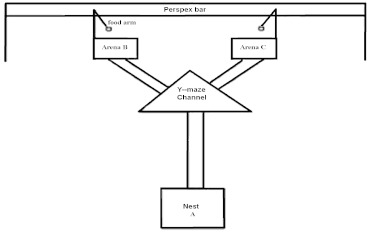
Diagrammatic representation of the Y-maze device used for the study of recruitment pattern in *Microcerotermes fuscotibialis.* High quality figures are available online.

This study is aimed at investigating the ability of termites to communicate changes in food supply with a view to determining the role of food in regulating the pattern of recruitment in *M. fuscotibialis.*

## Materials and Methods

Three arboreal Colonies of *M. fuscotibialis* were collected from the trunks of *Mussaenda elegans* Schumacher and Thonning (Rubiales: Rubiaceae), located within a radius of 100 m on the campus of University of Lagos, Nigeria. These colonies were kept in the laboratory for at least seven days before use at temperatures of 27 ± 3° C and 70% RH. Artificial nests were then made out of these field colonies.

### Experimental set-up

The experimental set-up consists of an artificial nest (A) connected to the stem base of a Y- channel and two silt-coated arenas-B and -C attached to each arm of the channel (Figure 1). The inner covers of the two arenas carried respectively, a cut piece of sugar cane (20 mm × 10 mm), herein referred to as “food”, affixed to each end of two pins. One end of a nylon thread was thereafter tied to these food-carrying pins, while each of its free ends was attached to two other pins, carrying two fairly large plastic corks (which served as weights for suspension). By raising and lowering the suspended weights over the Perspex bar, the food could be raised or lowered into either of the two arenas without disturbing the experimental set-up. All experiments were replicated five times, while the mean value obtained compared using the Student-t test at 0.05 level of significance. The t-statistics follows the following assumptions: H_o_: µ^1^-µ^2^ = 0 and H_A_: µ^1^-µ^2^ ≠ 0 at n_1_+n_2_ -2 degrees of freedom (df). When the calculated t-test statistic, t_c_ is greater than the tabulated t_t_, then significant difference between the two means is assumed, while if t_c_ is less than t_t_, then there is no significant difference between the two tested mean traffic.

### 1) Termite traffic regulation to soil-food arenas

The termites were allowed to carry on their normal foraging activities, after which food was lowered into one of the arenas (B-arena), while observations on termite traffic situation into the C-arena (which contained soil) were made. At the end of 30 minutes, food was raised from B-arena and simultaneously lowered into C-arena, and observation of termite's movement into this arena (C) continued for the next 30 minutes.

### 2) Termite traffic regulation to food-soil arenas (Food Reversal)

The effect of food reversal on termite traffic to food-soil choice situation was investigated. After the normal termite foraging activities into the arenas, food was lowered into Barena, while C-arena contained soil coating. Termite traffic into the food arena (B) was recorded for 30 minutes. Thereafter food was raised from the B-arena, and simultaneously lowered into C-arena, while observations continued on the B-arena for another 30 minutes.

The next stage of this study investigated the effect an additional food source would have on recruitment activities of termites towards an already existing food source and vice versa.

### 3) Effect of an additional food source on termite traffic towards an already existing food source.

Food was lowered into C-arena, while B-arena contained soil. Observation on termite traffic to and from C-arena was carried out for 30 minutes. Thereafter, food was lowered into Barena without raising the food in the C-arena. Observation continued on C-arena for another 30 minutes.

### 4) Effect of an already existing food source on termite traffic towards a new food source.

Similarly, food was initially lowered into Barena, while C-arena contained soil. Observation on termite movement into the soil arena-C was made for 30 minutes. At the end of this period, food was lowered into C-arena without raising the food in B-arena. Observation on C-arena continued for the next 30 minutes.

## Results

### 1) Termite traffic regulation to soil-food arenas

**Figure 2.**
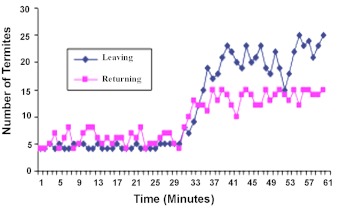
Traffic of termites into soil-food arenas. Mean of 5 replicates. Food in arena-B, Soil in arena-C. Initial observation for 30 min of arena-C. Food was relocated from arena-B into arena-C. Observation continued of arena-C for the next 30 min. High quality figures are available online.

The pattern of traffic to the soil arena-C showed that the number of outgoing termites (referred to as outgoers) fluctuated within 4 to 5 termites per minute, while the returning termites (herein referred to as returners) moved into the nest at the rate of 4–8 termites per minute throughout the 30 minute duration of the first stage of this study (Figure 2). Observation further revealed movement into this arena to be slow and sluggish. A mean total population of 133 ± 3 (SD) outgoers moved from the nest to the soil arena, while 172 ± 3.16 (SD) returners moved out of the soil arena to the nest. However, when food was raised from B-arena and simultaneously lowered into the C- arena (now containing food), the following activities was observed: between the 31^st^ and 34^th^ minute, the number of outgoers ranged from 7 to 12 termites per minute. And from the 35^th^ to 50^th^ minute, the outgoers maintained a high traffic of 15–23 termites per minute. This increased activities continued into the 60^th^ minute, with the termites moving at the rate of 15–25 termites per minute. A total population of 571 ± 4 (SD) outgoers moved into the food arena.

In testing the difference between the mean traffic of outgoers to the soil and food arenas, the results showed that there was a significant traffic movement into the food arena (t_c_>t_t_) at 0.05 level of significance: t_c_= 184.48; t_t_(8df) = 2.306.

For the returners, termite movement from the arena to the nest ranged from 8 to 13 termites per minute between the 31^st^ and 34^th^ minute. From the 35^th^ to 50^th^ minute, movement of returners fluctuated within 10 and 15 termites per minute. This rate was maintained up to the 60^th^ minute (Figure 2). A mean total population of 396 ± 3.16 (SD) returners moved from the food arena back to the nest. A comparative analysis of the mean returning traffic under food and non food situation showed that there was a significant increase in traffic from the food arena compared to when the arena contain soil (t_c_ > t_t_); t_c_ = 224; t_t_ (8df) = 2.306 at 0.05 level of significance.

These results therefore showed that there was quantitative recruitment into C-arena as soon as the scouting foragers detect food. Increase in termite traffic into the new food arena was noticeable as from the fourth minute after food was lowered into the arena.

### 2) Termite traffic regulation to food-soil arenas (Food Reversal)

**Figure 3.**
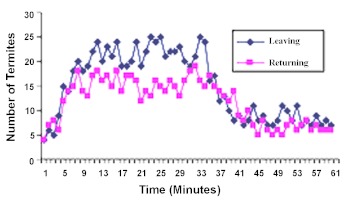
Traffic of termites into food-soil arenas. Mean of 5 replicates. Soil in arena-B, food in arena-C. Initial observation for 30 min of arena-C. Food was relocated from arena-C into arena-B. Observation continued of arena-C for the next 30 min. High quality figures are available online.

The effect of food removal on termite traffic is shown in Figure 3. Initially when the Carena was supplied with food, traffic of outgoers ranged from 4–9 termites per minute within the first four minutes. Between the 5^th^ and 20^th^ minute, the outgoing population fluctuated within 14–24 termites per minute. The rate of movement of the outgoers between the 21^st^ and 30^th^ minute ranged within 19–25 termites per minute. A mean total of 562 ± 2.92 (SD) outgoing traffic moved into the food arena.

For the returning population, the rate of movement of termites within the first four minutes was 13–15 termites per minute. From the 5^th^ to 20^th^ minute, the number of returning termites ranged between 12–18 termites per minute. Between 21^st^ and 30^th^ minutes, the returners moved at the rate of 12–16 termites per minute, with a mean total of 420 ± 2.35 (SD) termites (Figure 3).

These results showed an increase in the rate of activities of these termites towards the food arena. However, when food was raised from B-arena, the following observations were made: the outgoing termites moved at the rate of 19–25 termites per minute within the first four minutes after food removal. Between the 35^th^ and 50^th^ minutes, movement of the outgoers declined from 17 to 7 termites per minute. As from the 51^st^ to 60^th^ minute, movement of the outgoers fluctuated within 7–11 termites per minute. A reduced termite traffic was recorded, totaling 332 ± 3.41 (SD) termites. This reduction in outgoing traffic was significant (t_c_>t_t_; t_c_ = 106.48; t_t_ (8df) = 2.306) following the removal of food from the arena.

The returning population moved at the rate of 15 to 19 termites per minute between the 31^st^ and 34^th^ minutes, while the rate declined from 17 to 5 termites per minute between the 35^th^ and 50^th^ minutes. Rate of movement of these returners fluctuated within 6 and 8 termites per minute between the 51^st^and 60^th^ minutes (Figure 3). The returning population averaged 289 ± 3.54 termites. This reduction in termite traffic was also significant (t_c_>t_t_; t_c_= 68.95; t_t_ (8df) = 2.306), after removal of food from the arena.

One noticeable feature of this aspect of study was a marked reduction in the activities of these termites within 4–7 minutes after food removal. This indicates that spatial information was passed on to other comrades in the nest on the food situation in the arena, resulting in reduction in the number of termites visiting the former food arena-B.

**Figure 4.**
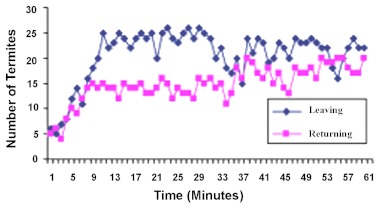
Effect of a new food source on termite traffic into an old food arena. Mean of 5 replicates. High quality figures are available online.

**Figure 5.**
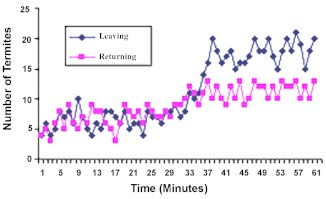
Effect of an old food source on termite traffic into a new food arena. Mean of 5 replicates. High quality figures are available online.

In the second stage of this work, Figures 4 and 5 shows the results obtained from the response of termite population to the influence of an additional food source in relation to an already existing one.

### 3) Effect of an additional food source on termite traffic towards an already existing food source.

When B-arena initially contained soil and the C-arena contained food, movement of termites into C-arena fluctuated between 5–8 termites per minute in the first four minutes. From the 5^th^ to 20^th^ minute, termite movement into Carena ranged from 11 to 25 termites per minute, while the rate fluctuated between 20 and 26 termites per minute between the 21^st^ and 30^th^ minute (Figure 4). A mean total traffic of 600 ± 16.96 (SD) outgoers moved into this arena.

The returning termite population maintained a fluctuating range of 4 to 8 termites per minute within the first four minutes, while a range of 9 and 15 termites per minute observed between the 5^th^ and 20^th^ minute. From the 21^st^ to 30^th^ minutes, termite traffic returning from the food arena to the nest fluctuated between 12 and 16 termites per minute (Figure 4). A total of 376 ± 20.74 (SD) of returning traffic were involved.

The addition of a new food source (in Barena) however, did not change the pattern of recruitment activities towards the old food arena. Outgoing traffic throughout the 30 minute duration was maintained within 15 and 24 termites per minute, with a total population of 615 ± 16.58 (SD). The returners moved at the rate of 11 and 20 termites per minute over the same period of time, with a population of 508 ± 34.07 (SD) (Figure 4). Traffic to the old food arena was not significantly affected by the presence of a new food source (t_c_ <t_t_)_;_ t_c_ = 1.41; t_t_ (8df) = 2.306 at 0.05 level of significance.

### 4) Effect of an already existing food source on termite traffic towards a new food source.

Similarly, when B-arena was initially supplied with food, the traffic of outgoers into the soil arena-C in the first 30 minutes ranged within 4–10 termites per minute, with a mean total of 196 ± 1.58 (SD) termites. The returning population over the same period moved at the rate of 3–9 termites per minute, totaling 202 ± 2.92 (SD) (Figure 5). However, the traffic situation changed when food was lowered into C-arena, despite the presence of food in B-arena. Outgoing termite population into arenaC within the first four minutes ranged from 7 to 11 termites per minute. Between the 35^th^ and 50^th^ minutes, the outgoing traffic fluctuated within 11 and 20 termites per minute. From the 51^st^ to 60^th^ minutes, the outgoers moved at the rate of 15–21 termites per minute. An average of 487 ± 3.54 (SD) termite moved from the nest into the new food arena. These results indicated that, even though there was a significant increase in termite traffic into this new food arena-C, the pre-existing old food arena did not have any significant impact on termite traffic to the new food arena (t_c_ <t_t_); t_c_ = -169.94; t_t_ (8df) = 2.306, at 0.05 level of significance.

For the returners, the termites moved at the rate of 9–12 termites per minute between the 31^st^ and 34^th^ minutes. From the 35^th^ to 50^th^ minutes, the returning traffic fluctuated within 9 and 13 termites per minute. Between the 51^st^ and 60^th^ minutes, the returners moved at the rate of 10–13 termites per minute (Figure 5). A total average of 334 ± 3.24 returners returned to the nest from the food arena. Subjecting these results to the t-statistic analysis, showed that the old food arena did not have any significant effect on the traffic population of returners from the new food arena (t_c_ <t_t_); t_c_ = -67.69; t_t_ (8df) = 2.306, at 0.05 level of significance)

These results therefore showed that the effect of an additional food source on recruitment activity in *M. fuscotibialis* did not become dominant over the operating effect of an already existing food source, and vice versa.

## Discussion

This study has shown that food plays a major role in determining the pattern of recruitment in *M. fuscotibialis.* The observations show that regulation of termite traffic was effected within four to seven minutes through a rise or drop in traffic population when food was introduced or removed from the arena. Similar observations were made by Oloo and Leuthold (1979), in foraging population of *Trinervitermes bettonianus*, and Olugbemi and Malaka (2007) in *M. fuscotibialis*, where the detection of food stimulates workers to lay stronger “recruitment trail” to motivate nest mates to mass foraging.

It could therefore be inferred that the recruitment trail produced by workers of *M. fuscotibialis* could either be reinforced (when food was present in the arena) or ceases to be laid (when food was removed from the arena). This agrees with the findings of Traniello (1981), who observed that the trail pheromone of *Nasutitermes coastalis* was characterized by an ephemeral component that regulates recruitment, and an incredibly persistent substance that provides a long lasting orientation cue. Similar observations were made by Malaka and Leuthold (1986), on *Amitermes evuncifer*, and Traniello et al. (1994), on *Reticulitermes* spp.

Observations by Andara et al. (2004) also revealed a novel recruitment strategy in the termites, *Nasutitermes ephratae* and *N. corniger*, in which an “autocratic decision making system” was utilized during their recruitment process, meaning that a single or few termite scouts can provide the colony all the information required to initiate and maintain chemical recruitment to food sources.

This “autocratic” recruitment strategy may be largely operational in the recruitment activities of *M. fuscotibialis.*

This study has also shown that the effect of a newly supplied food source did not become dominant over an already existing one, with the traffic population being shared between the old and new food sources. This indicates that the strong recruitment trail towards the old food arena did not become weaker, despite the laying of new trails and recruitment of other nest mates towards a new food source. This is another classical case of “autocratic” decision making mechanism operating, since the trail towards the new food source did not divert termite traffic from the existing food source to the new one.

## Conclusions

It has been established from this study that food plays a major role in the recruitment activities in *M. fuscotibialis*, and when this (food) is detected, information about its location was quickly communicated to other confederates, which were subsequently recruited to the food site. Removal of this food also brings about a significant reduction in termite traffic to the food arena.

Recruitment patterns in this termite towards an old food source were not affected by the presence of an additional food source, nor was the effect of this new food source dominant over the existing one.

Recruitment decision mechanism may be largely “autocratic” in the recruitment process of this termite species.

## References

[bibr01] Andara C, Issa S, Jaffe K (2004). Decisionmaking Systems in Recruitment to Food for two Nasutitermitinae (Isoptera: Termitidae).. *Sociobiology*.

[bibr02] Campora E, Grace JK (2001). Tunnel orientation and search pattern sequence of the formosan subterranean termite (Isoptera: Rhinotermitidae).. *Journal of Economic Entomology.*.

[bibr03] Hedlund JC, Henderson G (1999). Effect of available food size on search tunnel formation by the Formosan subterranean termite (Isoptera: Rhinotermitidae).. *Journal of Economic Entomology*.

[bibr04] Jaffe K (1980). A theoretical analysis of the communication system for chemical mass recruitment in ants.. *Journal of Theoretical Biology.*.

[bibr05] Jaffe K, Villegas G, Colmenares O, Puche H, Zabala N, Alvarez M, Navarro J, Pino E (1985). Two different decision-making systems in recruitment to food in ant societies.. *Behaviour.*.

[bibr06] Kaib M., Gribakin FG, Wiese K, Popov AV (1990). Intra- and interspecific chemical signals in the termite, *Schedorhinotermes-* production sites, chemistry and behaviour.. *Sensory Systems and communication in Arthropods: Advances in Life Sciences*.

[bibr07] Malaka SLO, Leuthold RH (1986). Mechanisms of recruitment for the retrieval of food in *Amitermes evuncifer* Silvestri (Isoptera: Termitidae: Termitinae).. *Insect Science and Application.*.

[bibr08] Oloo GW, Leuthold RH (1979). The Influence of food on trail laying and recruitment behaviour in *Trinervitermes bettonianus* (Termitidae: Nasutitermitinae).. *Entomologia Experimentalis and Applicata.*.

[bibr09] Olugbemi BO, Malaka SLO (1994). Effect of food on recruitment activities in *Microcerotermes fuscotibialis* Sjostedt, 1896 (Isoptera: Termitidae: Termitinae).. *Journal of Scientific Research and Development.*.

[bibr10] Olugbemi BO, Malaka SLO (2007). The effect of food on pheromonal communication in the termite. *Microcerotermes fuscotibialis* Sjostedt *African Journal of Ecology.*.

[bibr11] Robson SK, Lesniak MG, Kothandapani RV, Traniello JFA, Thorne BL, Fourcassie V (1995). Non-random search geometry in subterranean termites.. *Naturwissenchaften.*.

[bibr12] Traniello JFA (1981). Enemy deterrence in the recruitment strategy of a termite soldier organized foraging in *Nasutitermes costalis*.. *National Academy of Science, U.S.A.*.

[bibr13] Traniello JFA, Buscher C (1985). Chemical regulation of polyethism during foraging in the Neo-Tropical termite, *Nasutitermes costalis*.. *Journal of Chemical Ecology.*.

[bibr14] Traniello JFA, Robson SK, Lesniak M, Kothandapani R (1994). Organization of searching in subterranean termites, *Reticulitermes* spp..

[bibr15] Wilson EO (1962). Chemical communication among workers of the fire ant *Solenopsis saevissima*.. *Animal Behaviour.*.

